# A Case of Congenital Atelectasis

**Published:** 1893-05

**Authors:** Alphonse Dagenais

**Affiliations:** 473 Virginia Street; Buffalo, N. Y.


### BUFFALO ACADEMY OF MEDICINE.

*Stated Meeting, held Tuesday, March 21, 1893.*

Reported by WILLIAM C. KRAUSS, M. D., Secretary.

DeLancey Rochester, M. D., President, called the meeting to order at 8.30 P. M., when the following papers were read and discussed:

### A CASE OF CONGENITAL ATELECTASIS.

By ALPHONSE DAGENAIS, M. D., Buffalo, N. Y.

Being requested to present a very short paper before this section of the Academy of Medicine, I have selected the report of a case of congenital atelectasis, which occurred recently in my practice, it being the first case of this nature that I have been called upon to witness. This, or better, the atelectatic condition of the lungs, I have met several times, especially in whooping cough, where stimulated treatment generally overcame the difficulty; but never have I witnessed the symptoms of atelectasis so general, so pronounced, as in this case. I still hesitated to report the case, because of not being able to tell the appearance of the lungs after death, a post-mortem having been refused as usual.

Mrs. G. was delivered of an apparently very healthy child during the month of February last. To all appearances the child seemed strong and well-developed, the labor had been natural, without artificial or instrumental help. In spite of this splendid physical appearance, the child lay quietly, without attempting any muscular movements, and his demeanor indicated a lack of vigor. He never cried, had only piteous moans, most of the time scarcely audible. After the second day he could move his arms, limbs, and head, showing no spinal affections or deformities, no abdominal tumor could be detected which would cause a displacement upward of the diaphragm, no vesicular murmur could be heard with a stethoscope at the posterior and anterior portion of the lungs. The lungs seemed collapsed, airless. A sort of gurgling sound was mostly constantly heard, whether awake or asleep. On about the fifth day the cyanotic condition, observed from birth, became more pronounced; the surface, especially the face, the fingers, and the feet, became cyanotic and the extremities cold. The temperature was normal, but the pulse feeble. The breathing was fast and shallow,—fifty to sixty. Most of the time was passed in sleep. He could hardly nurse. This life lasted fifteen days. Muscular twitchings warned us of the approaching comatose condition, slight convulsions preceded the fatal termination.

We read that congenital atelectasis is not commonly due to any vice or disease of the pulmonary organs, but is produced by any condition which prevents the prompt establishing of the functions of the respiration. In this case it was, no doubt, the result of causes which had been in operation during the intrauterine life of the child, for there was no physical weakness, no premature birth, no compression of the cord—in fact, no common causes of atelectasis. Intracranial effusions might have brought pressure upon the pneumogastric. We are told that when a large tract of lung is disabled, as in the case related, important changes ensue in the organs of circulation. The impediment of the movement of the blood through the lungs results in stasis in the pulmonary artery and the entire venous system, and leads to hemorrhagic tendencies and edema of the unaffected lung tissue. The same condition also tends to prevent, in congenital cases, the closure of the fetal channels of circulation, especially the foramen ovale, which explain the cyanotic condition of the child.

The treatment in this case was, first, the cutting of the cord without ligature, allowing a certain amount of blood to escape; then hot immersions of the chest with water, to which a small addition of mustard was added ; then alcoholic applications were made, woolen wrappings, minute doses of ipecac, with the expectation of expelling viscid phlegm from the bronchial tubes and producing respiration, but all without any good result.

473 VIRGINIA STREET.

